# Editorial: Current and future role of artificial intelligence in cardiac imaging, volume II

**DOI:** 10.3389/fcvm.2023.1220836

**Published:** 2023-06-08

**Authors:** Tim Leiner, Karim Lekadir, Steffen E. Petersen, Alistair A. Young

**Affiliations:** ^1^ Department of Radiology, Mayo Clinic, Rochester, MN, United States; ^2^ Departament de Matemàtiques and Informàtica, Universitat de Barcelona, Artificial Intelligence in Medicine Lab (BCN-AIM), Barcelona, Spain; ^3^ Barts Heart Centre, Barts Health NHS Trust, London, United Kingdom; ^4^ NIHR Barts Biomedical Research Centre, William Harvey Research Institute, Queen Mary University of London, London, United Kingdom; ^5^ School of Biomedical Engineering and Imaging Sciences, King’s College London, London, United Kingdom

**Keywords:** artificial intelligence, cardiac imaging modalities, big data, cardiac image analysis, cardiovascular personalized medicine, AI adoption and translation

**Editorial on the Research Topic** Current and future role of artificial intelligence in cardiac imaging, volume II

## Introduction

Heart imaging without the need for invasive procedures plays a crucial role in various aspects of cardiology, including diagnosis, risk evaluation, treatment decisions, medical and invasive therapies, prognosis, and ongoing monitoring. As a key component in the pursuit of precision medicine, cardiac imaging enables a personalized approach to healthcare. Cardiac imaging is essential for a comprehensive understanding of cardiovascular conditions, intricate physiology, and the implications of imaging results for managing cardiovascular health and disease. Consequently, the demand for cardiac imaging continues to grow.

Artificial Intelligence (AI) methods are increasingly needed for improving imaging exams, to optimize workflow, reduce waiting times, improve accuracy and precision, reduce costs and benefit patient outcomes. The special issue on “Current and Future Role of Artificial Intelligence in Cardiac Imaging” in 2020 provided 10 comprehensive reviews of a wide range of AI applications in cardiovascular imaging, from efficient image acquisition and fast reconstruction, to structure and function analysis, to statistical atlases and imaging-genetics synergies. In the meantime the field has been advancing rapidly. The second volume of this topic focuses on original research articles which showcase new methods and applications. Applications were invited for all topics and forms of cardiac imaging, and the resulting published papers reflect a range of modalities and applications which have a common goal of improving patient outcomes through extraction of more detailed and precise information than has previously been possible.

## Special issue content

**Figure 1:**
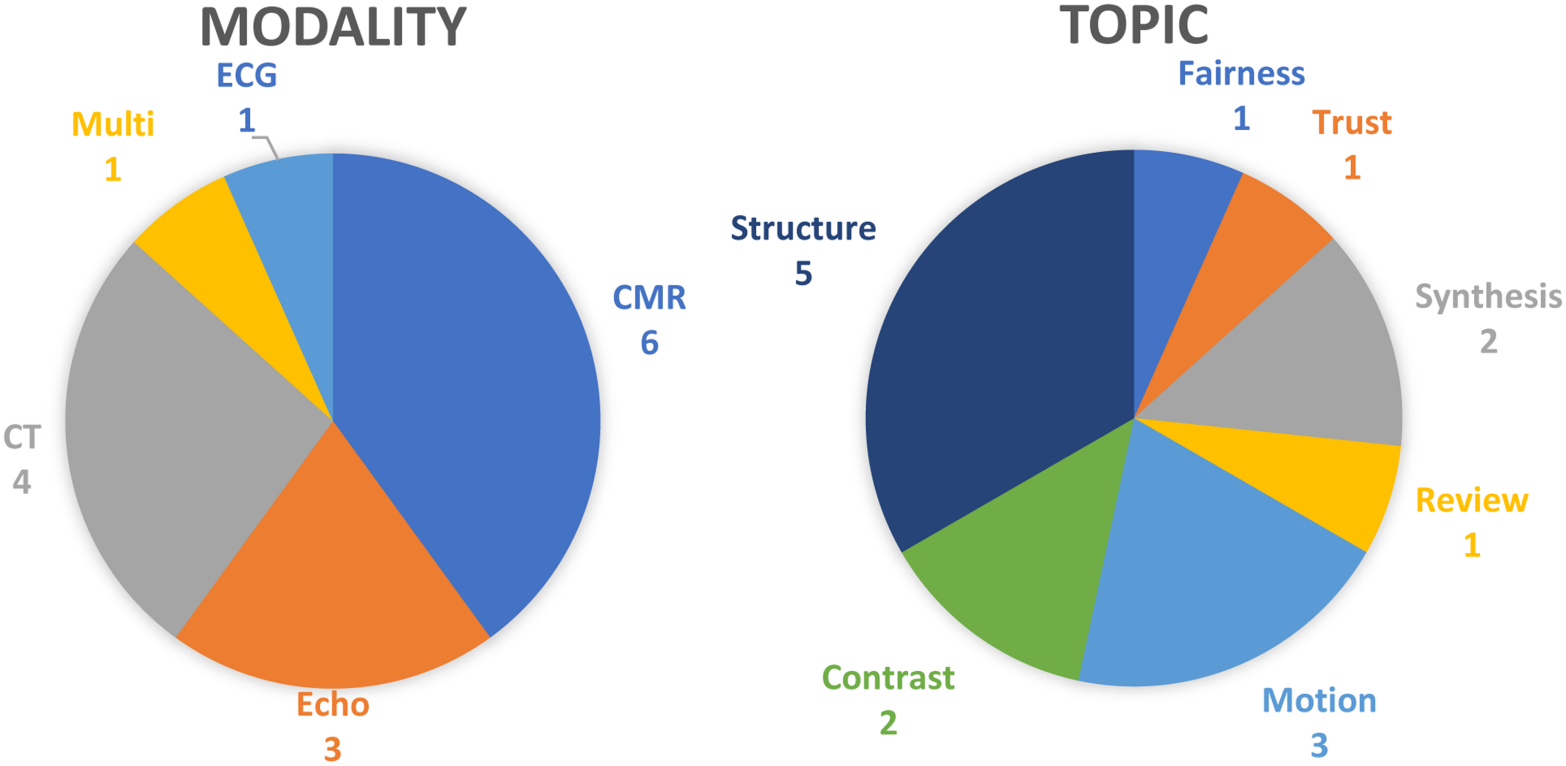
Breakdown of special issue papers by modality and topic.

The special issue attracted a greater number of submissions than the first edition, with 15 papers eventually published (Abdulkareem et al., Alabed et al., Beetz et al., Campello et al., Chen et al., Hampe et al., Li et al., Lin et al., Lin et al., Puyol-Antón et al., Suinesiaputra et al., Szabo et al., Zhai et al., Zhao et al. and Zhao et al.). Figure 1 shows a breakdown of topics and applications.

Imaging modality was varied with CMR being the most common (Abdulkareem et al., Alabed et al., Beetz et al., Campello et al., Puyol-Antón et al. and Suinesiaputra et al.), followed closely by CT (Chen et al., Hampe et al., Li et al. and Zhai et al.) and echocardiography (Lin et al., Lin et al. and Puyol-Antón et al.). One paper provided a guide to trustworthy and responsible AI in all imaging modalities (Szabo et al.). Although not an imaging method, ECG processing is an important topic in cardiovascular disease, and can give anatomical information such as hypertrophy, which was the focus of one paper (Zhao et al.). In terms of application area, the most common was structural analysis (segmentation of chambers, vessels, and detection of hypertrophy) (Hampe et al., Lin et al., Suinesiaputra et al., Zhao et al. and Zhao et al.), followed by image motion analysis [in echo (Lin et al.) and CT (Chen et al. and Li et al.)]. These reflect the ongoing development of methods for quantifying anatomy and function in cardiac images, which are extremely important for diagnosis and prognosis. Two papers were concerned with synthesis of images or anatomy, using generative adversarial networks (Campello et al.) and variational auto encoders (Beetz et al.) respectively. This is an exciting area of research with the promise to synthesise large numbers of datasets with different pathology and characteristics, which can be used to understand the influence of disease processes on structure and function. Two papers discussed learning contrast-enhanced information from non-contrast scans, applied to late gadolinium enhancement CMR (Abdulkareem et al.) and calcium scoring CT (Zhai et al.) respectively. These highlight the ability of AI methods to leverage information present in standard scans to provide additional information, which would normally require an extra scan and administration of a contrast agent. The papers on AI fairness (Puyol-Antón et al.), and trustworthiness (Szabo et al.), illustrate another promising area of research into methods for improving AI methods, mitigating bias in the training data and explaining how inferences are made in complex networks. These were identified in the first special issue as topics of high importance. Another important topic highlighted by the first special issue is the reporting of AI methods to foster reproducibility and generalization, and this is the focus of the review article (Alabed et al.).

## Future perspectives

AI methods are becoming ubiquitous in all areas of cardiac imaging. This is driven by the increasing role of imaging in guidelines for patient treatment, as well as the wealth of data generated by large cohort imaging studies such as the UK Biobank and the Multi-Ethnic Study of Atherosclerosis. The recent advent of widely available large language models which link imaging and videos with text such as GPT-4 highlight the rapidly advancing technology which will transform medical imaging in general and cardiovascular imaging in particular. The ability to generate reports from images and to combine images and reports into structured datasets will enable large amounts of data already residing in hospital databases to be reused for new applications. Generative models will enable new understanding of the ways in which longitudinal changes between imaging exams can be more precisely quantified, by comparing predicted follow-up images with the actual scans and highlighting any anomalies. Physics-based networks and neural implicit functions are also on the horizon which will enable super-resolution in fluid flow imaging, and computation of physical entities such as pressure and stiffness from imaging data. The linking of digital twins with imaging methods will enable surrogate AI-based methods to simulate growth and remodeling in health and disease, to estimate physiological parameters from non-invasive imaging. In summary, this second issue on AI in cardiac imaging illustrates important areas which are rapidly developing. However the next advances in this area are only limited by the imaginations of the researchers in the community. At present, there is no AI solution for this!

